# Supplementary material for: Causal Relationship between Aging and Anorexia Nervosa: A White-Matter-Microstructure-Mediated Mendelian Randomization Analysis
Source: Biomedicines. 2024 Aug 16;12(8):1874. doi: 10.3390/biomedicines12081874 (PMC11351342; doi:10.3390/biomedicines12081874)
Supplement: Supplementary file 1 [file biomedicines-12-01874-s001.zip › Supplementary Figure/Figure S4/Singe SNP plot of MR analysis for TL to ubm-b-1941.pdf]

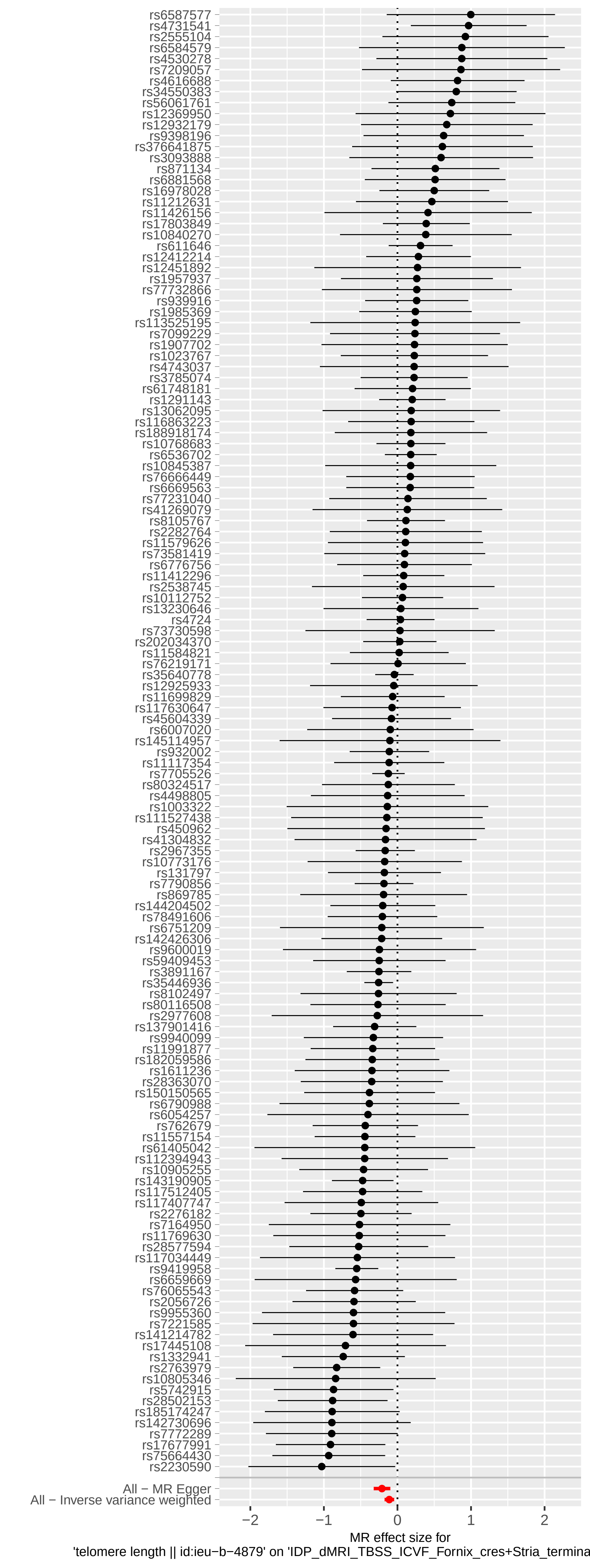
rs6587577
rs4731541
rs2555104
rs6584579
rs4530278
rs7209057
rs4616688
rs34550383
rs56061761
rs12369950
rs12932179
rs9398196
rs376641875
rs3093888
rs871134
rs6881568
rs16978028
rs11212631
rs11426156
rs17803849
rs10840270
rs611646
rs12412214
rs12451892
rs1957937
rs77732866
rs939916
rs1985369
rs113525195
rs7099229
rs1907702
rs1023767
rs4743037
rs3785074
rs61748181
rs1291143
rs13062095
rs116863223
rs188918174
rs10768683
rs6536702
rs10845387
rs76666449
rs6669563
rs77231040
rs41269079
rs8105767
rs2282764
rs11579626
rs73581419
rs6776756
rs11412296
rs2538745
rs10112752
rs13230646
rs4724
rs73730598
rs202034370
rs11584821
rs76219171
rs35640778
rs12925933
rs11699829
rs117630647
rs45604339
rs6007020
rs145114957
rs932002
rs11117354
rs7705526
rs80324517
rs4498805
rs1003322
rs111527438
rs450962
rs41304832
rs2967355
rs10773176
rs131797
rs7790856
rs869785
rs144204502
rs78491606
rs6751209
rs142426306
rs9600019
rs59409453
rs3891167
rs35446936
rs8102497
rs80116508
rs2977608
rs137901416
rs9940099
rs11991877
rs182059586
rs1611236
rs28363070
rs150150565
rs6790988
rs6054257
rs762679
rs11557154
rs61405042
rs112394943
rs10905255
rs143190905
rs117512405
rs117407747
rs2276182
rs7164950
rs11769630
rs28577594
rs117034449
rs9419958
rs6659669
rs76065543
rs2056726
rs9955360
rs7221585
rs141214782
rs17445108
rs1332941
rs2763979
rs10805346
rs5742915
rs28502153
rs185174247
rs142730696
rs7772289
rs17677991
rs75664430
rs2230590
All − MR Egger
All − Inverse variance weighted
−2
−1
0
1
2
MR effect size for
'telomere length || id:ieu−b−4879' on 'IDP_dMRI_TBSS_ICVF_Fornix_cres+Stria_termina
